# Obesity Cardiomyopathy as a Cause of Sudden Cardiac Death: Heavy Matters∗

**DOI:** 10.1016/j.jacadv.2023.100434

**Published:** 2023-07-28

**Authors:** Timothy P. Fitzgibbons

**Affiliations:** Cardiovascular Division, Department of Medicine, University of Massachusetts Chan School of Medicine, Worcester, Massachusetts, USA

**Keywords:** cardiac magnetic resonance imaging, echocardiography, left ventricular hypertrophy, obesity, sudden cardiac death

**Figure:**
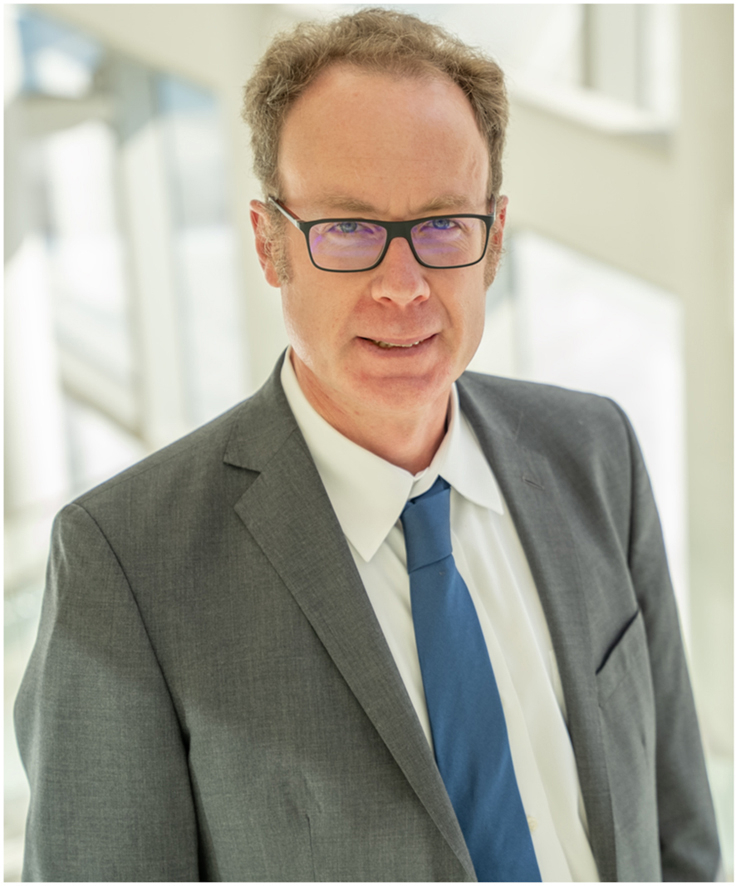

In this issue of *JACC: Advances*, Westaby et al^1^ present a case-control study from the Cardiac Risk in the Young Center for Cardiovascular Pathology at St. George’s University in London, UK. The center was referred 6,457 cases of sudden cardiac death (SCD), of which 4,492 had a recorded body mass index (BMI). 1,202 of the 4,492 patients were obese (BMI ≥30 kg/m^2^) and 3,290 were normal or overweight (BMI ≤30 kg/m^2^). The authors defined cardiomegaly as a heart weight above 450 g in women and 550 g in men; a definition based on guidelines published by the Association for European Cardiovascular Pathology.^2^ Cases with coronary artery disease, hypertrophic cardiomyopathy, hypertension, or valvular heart disease were excluded. In obese subjects, there were 53 cases of unexplained cardiomegaly and in normal weight subjects there were 14 cases. The odds ratio for cardiomegaly in obesity was 5.3 (95% CI: 2.9-9.6; *P* < 0.001). Subjects who were obese and had cardiomegaly were defined as obesity cardiomyopathy (OCM).^1^ OCM cases had a mean age of 42 ± 12 years, were mostly men (64%) with a mean BMI of 42 ± 8 kg/m^2^ and a mean heart weight of 598 ± 93 g. In comparison to matched normal weight and obese controls, the OCM group had increased epicardial fat and uniformly greater left ventricular (LV) wall thickness. All OCM subjects had myocyte hypertrophy, but only 13% had fibrosis on histology, and none had myocyte disarray.^1^

This study has important implications considering the global burden of obesity and the socioeconomic consequences of the loss of these young productive members of society. The prevalence of obesity in children continues to rise across the globe. In 1975, only 4% of children aged 5 to 19 were overweight or obese and in 2016 18% of children were overweight or obese.^3^ It is very difficult to lose and maintain weight once it is gained and obese children are likely to be obese adults.^4^ Obesity in childhood is associated with a greater risk and earlier onset of cardiovascular disease and diabetes. One in 5 adults in the world is expected to be obese by 2025.^3^

This report adds to a body of literature suggesting that left ventricular hypertrophy (LVH) due to obesity is a risk factor for SCD.^5^^,^^6^ A recent paper from Australia compared obese vs nonobese SCD victims ages 18 to 50 years.^6^ Although they did not exclude subjects with comorbid conditions, only 10% of victims with a BMI >50 kg/m^2^ had coronary disease while two-thirds had LVH. The definition of LVH in that study was similar; 400 g in females or 500 g in males in the presence of any wall diameter >15 mm.^6^

The strength of the current study is that subjects were younger and coexistent conditions were excluded.^1^ In the past, the adverse cardiovascular risk associated with obesity was attributed to comorbidities such as diabetes mellitus, sleep apnea, and metabolic syndrome. However, we know that obesity imparts a strong and independent risk of incident heart failure.^7^ The paper suggests that morbid obesity also imparts an independent risk of SCD, particularly in individuals with a BMI >35 kg/m^2^.^1^ If this is true, then what is the mechanism of SCD in OCM?

In Figure 2,^1^ the authors show the histology of 1 OCM case that has fatty infiltration of the left ventricle. They do not mention what percentage of cases had fatty infiltration of the right or left ventricle. Given that the OCM group had thicker epicardial fat pads, it is plausible that fatty infiltration of ventricular myocardium was present, as has been reported for the atrial myocardium.^8^ It is surprising that only 13% of OCM cases had fibrosis on histology, for this suggests that myocardial fibrosis was not the predominant cause of SCD and that late gadolinium enhancement would be absent in these patients if cardiac magnetic resonance imaging (MRI) were done for screening purposes. While some studies have shown fibrosis in LVH associated with obesity, others have shown little or none.^6^^,^^9^ Myocyte hypertrophy alone may result in abnormal depolarization and this is an important subject for future research. Obesity has been associated with an increased frequency of ventricular premature contractions at rest and during exercise.^10^^,^^11^ If LVH and myocyte hypertrophy are the triggers for SCD in obesity, what are the primary determinants of LVH?

In the absence of pathological conditions such as cardiomyopathy, volume or pressure overload, heart weight increases proportionally with weight, height, BMI, and body surface area.^12^ In normal subjects, body weight better predicts heart weight than height or BSA.^13^ Examination of Figure 4 suggests that a linear relationship exists between heart weight and body weight in the normal and obese groups but reaches an inflection point, or becomes nonlinear, at approximately a BMI of 35 kg/m^2^.^1^ It seems that near morbid obesity is associated with OCM, and perhaps, as the authors say, a BMI of 35 kg/m^2^ may be a threshold for the development of OCM. This raises the question of whether or not screening echocardiograms or MRIs should be recommended in younger patients at a certain BMI to identify those with OCM. If so, a practical definition or cutoff for OCM using left ventricular mass would be needed.

Practicing cardiologists are more familiar with estimates of left ventricular mass, not total heart weight. To put the findings of this study in context, consider the normal values for myocardial mass as measured by echocardiography and MRI.^14^^,^^15^ The upper limits of normal indexed LV mass by echocardiography are 88 g/m^2^ in women and 102 g/m^2^ in men.^15^ In cardiac MRI, the upper limits of normal are 68 g/m^2^ in women and 85 g/m^2^ in men. The limitations of both imaging modalities are beyond the scope of this paper but transthoracic echocardiogram is an overestimation and MRI is closer to the true value.^14^ Consider an average of the two, 78 g/m^2^ in women and 95 g/m^2^ in men. The left ventricle accounts for ∼55% of total heart weight at autopsy.^16^ Using this value, the estimated LV mass indices in the Westaby et al^1^ cohort were ∼100 g/m^2^ in normal and obese controls and ∼131 g/m^2^ in OCM. In addition to the increased mass, the wall thickness of the OCM group was high, suggestive of concentric LVH, a common finding in obese patients that has been validated by MRI.^12^^,^^17^

Interestingly, using cardiac MRI in the Dallas Heart Study, Khouri et al proposed a classification of hypertrophy that included both LV concentricity and end-diastolic volume. In the group of subjects with both concentric LV thickness and a dilated left ventricle, the mean BMI was 35 ± 7 kg/m^2^ and the estimated LV mass index was 150 ± 29 g/m^2^. The majority of these subjects were hypertensive males, but the similarity of these estimates to the OCM group in the present study is striking. One wonders if OCM is recapitulating this phenotype at a younger age and in the absence of hypertension.

In summary, this paper makes an important contribution to the literature that raises many important questions for future research. As the authors suggest, phenotype/genotype testing of cases and their families would be very informative to understand if there is any underlying genetic propensity for OCM.^1^ Basic science efforts focused on the electrophysiologic properties of hypertrophied cardiomyocytes might provide mechanistic insight into the etiology of SCD in obesity. Noninvasive screening for OCM should be considered in obese patients. Health systems need to be more aggressive in the identification and treatment of obesity in childhood and the medical and surgical treatment of severe obesity in young adulthood. Prevention efforts would be bolstered by legislation on a local and global scale that ensures: 1) everyone has access to healthy and affordable food options; 2) screen time is limited; and 3) physical activity is encouraged.

## Funding support and author disclosures

The author has reported that he has no relationships relevant to the contents of this paper to disclose.
